# 514. Anti-SARS-CoV-2 Monoclonal Antibodies for Early COVID-19: A Real World Experience

**DOI:** 10.1093/ofid/ofab466.713

**Published:** 2021-12-04

**Authors:** Katherine Belden, Bryan Hess, Caroline Brugger, Rachel Carr, Todd Braun, Joseph L DeRose, John Zurlo

**Affiliations:** 1 Sidney Kimmel Medical College at Thomas Jefferson University, Philadelphia, PA; 2 Thomas Jefferson University Hospital, Philadelphia, PA; 3 Abington Hospital-Jefferson Health, Abington, PA; 4 Temple University, Philadelphia, PA; 5 Jefferson University, Hershey, PA

## Abstract

**Background:**

Anti-SARS-CoV-2 monoclonal antibodies afford prompt immunity, have demonstrated reduction in severe COVID-19 in high risk ambulatory patients, and are available through Emergency Use Authorization. Challenges exist, however, to widespread utilization.

**Methods:**

This operations study 11/23/20-4/30/21 identified patients meeting monoclonal AB EUA criteria by test results or referral. Outreach to harder-hit neighborhoods included connecting with primary care teams and testing sites. Infusion centers with staff trained in infection control, rapid response and drug preparation were utilized. The primary study outcome was treatment of qualifying patients. Secondary outcomes included infusion complications, hospitalization/death, and symptom resolution. Investigational review board approval was obtained.

**Results:**

367 patients were treated: mean age of 63, 201(55%) male, 276(75%) white, 54(15%) black. All patients had a first positive direct SARS-CoV-2 test within 10 days, 232(63%) had > 1 high-risk qualification, 32(9%) were vaccinated for SARS-CoV-2. Of patients with available zipcodes, 135(38%) had a Community Need Index >3.5 and 157(45%) a Social Vulnerability Index >0.5. 190(52%) received bamlanivimab, 93(25%) casirivimab/imdevimab, 84(23%) bamlanivimab/etesevimab. Four patients experienced infusion reaction, 1 with anaphylaxis. 172(73%) of 236 patients were symptom free at day 5. 20 patients (5%) were hospitalized for COVID-19 within 30 days with a median time from symptom onset to infusion of 7 days, 11(55%) were admitted within 24 hours, 1 died.

Patient Characteristics

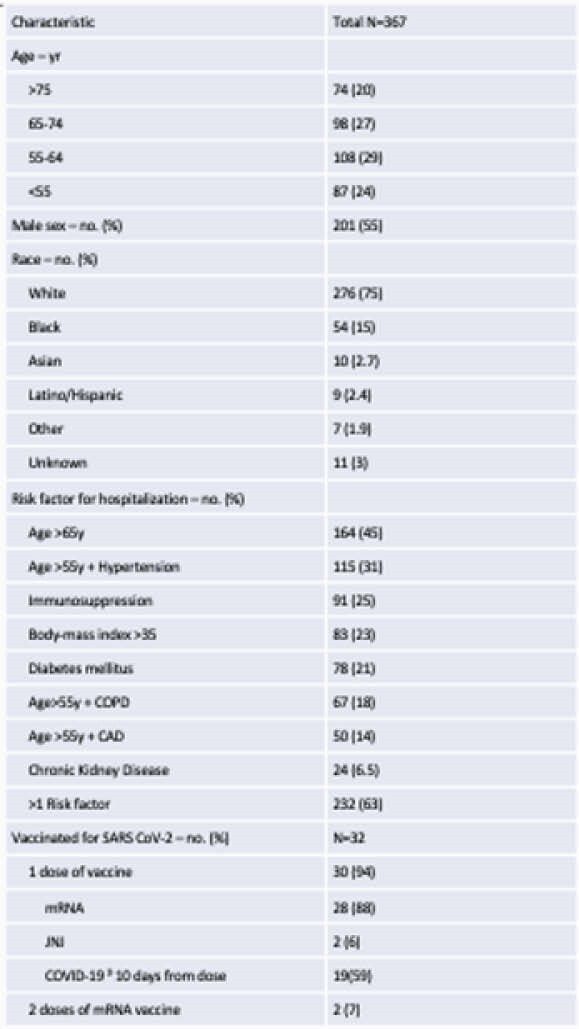

COVID-19 course

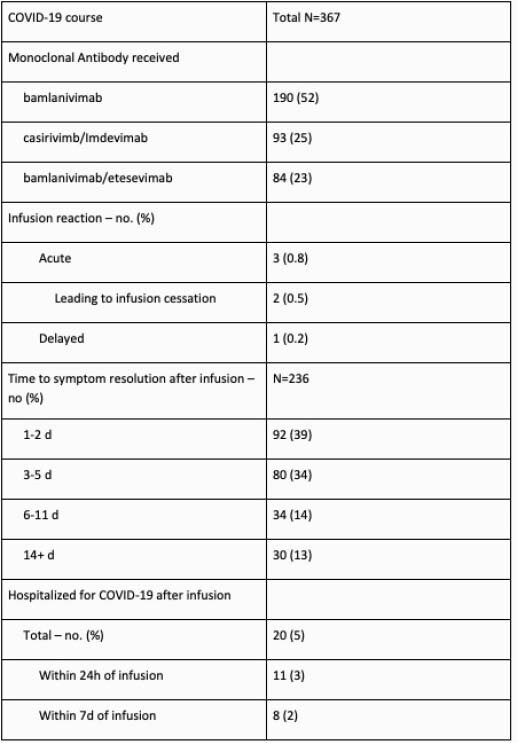

Community Need Index and Social Vulnerability Index by Zipcode

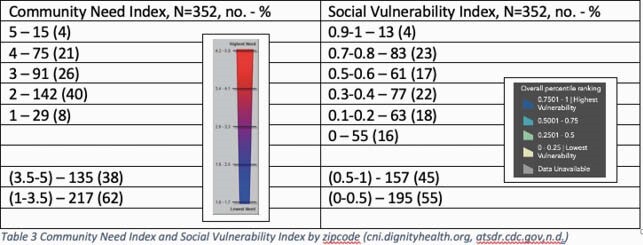

**Conclusion:**

Our study demonstrates that treatment with anti-SARS-CoV-2 monoclonal antibodies is feasible in a high resource setting. There were no related SARS-CoV-2 exposures and therapy was well tolerated. Trials of anti-SARS-CoV-2 monoclonal antibodies have reported lower rates of hospitalizations in treated patients than we found. This may reflect the expanded time frame for EUA therapy as compared to clinical trials, differences in real world patients or viral variants. Given potential benefit in unvaccinated patients or those at risk for poor vaccine response, the equitable utilization of anti-SARS-CoV-2 monoclonal antibody therapy in early COVID-19 should remain a focus for researchers and clinicians.

**Disclosures:**

**All Authors**: No reported disclosures

